# Skiing quality analysis of recreational skiers based on IMU data and self-assessment

**DOI:** 10.3389/fspor.2024.1495176

**Published:** 2024-12-24

**Authors:** Christina Kranzinger, Stefan Kranzinger, Eva Hollauf, Harald Rieser, Thomas Stöggl

**Affiliations:** ^1^ Human Motion Analytics, Salzburg Research Forschungsgesellschaft, Salzburg, Austria; ^2^ Red Bull Athlete Performance Center Salzburg, Salzburg, Austria

**Keywords:** alpine skiing, inertial measurement units, self-evaluation, skiing skills, sensor system

## Abstract

Alpine skiing is a popular sport in many countries and holds benefits in terms of health and well-being. At the same time alpine skiing is associated with a certain risk of accidents caused, among other things, by overestimating one’s own skiing skills. Self-assessment of skiing skills is not trivial. Therefore, feedback modalities can be assistive. One feasible option to provide skiers with feedback on their skiing ability are Inertial Measurement Units (IMUs). The aim of this study was to analyse the skiing quality of recreational skiers based on IMU data, collected with the Connected Boot sensor system with a living lab approach to investigate whether the skiing quality score delivers reasonable results for recreational skiers. The system has been developed with expert skiers and so far has not been validated with recreational skiers. Therefore, we aimed to investigate whether the objective skiing quality score of the Connected Boot corresponds to the self-assessed carving ability and to analyse changes in the assessment before and after the study. In total, data from 62 participants who skied with the sensor system were analysed. At the beginning and the end of the study the participants additionally received questionnaires to assess their skiing skills. The results show that there was a strong correlation between the self-reported carving ability and the skiing quality score of the Connected Boot and that the self-reported carving ability before the study was around 1.71 points higher than the algorithm-based skiing quality score. Interestingly, the correlation was higher for female compared to male participants.

## 1 Introduction

Alpine skiing is a popular sport in many countries. According to the report of Vanat (1) 68 countries in the world have at least one outdoor ski area equipped with lifts, summing up to a total number of 5,764 ski areas worldwide. In regular years between 350 and 380 million visits can be counted worldwide (1).

Alpine skiing has therefore substantial touristic and economic importance and has further positive health benefits with regard to cardiorespiratory and metabolic responses [see e.g., (2–4)] and well-being [see e.g., (5, 6)].

However, like other mountain sports, skiing is associated with a certain risk of accidents. For example, in Austria, 369 deaths on ski slopes were registered between the 2008/09 and 2017/18 winter seasons (7). Posch et al. (7) analysed that most of the accidents occurred while skiing (95.5%) and involved mainly men (87.3%). Also Dorsemaine et al. (8) concluded that male, young and highly qualified skiers in particular are victims of accidents caused by collisions with obstacles. Also with regard to fatalities in ski touring and freeriding in the years 2001 to 2019 in Berne (Switzerland), the proportion of male victims was with 80% many times higher than that of female victims (9). Luppino et al. (10) investigated Dutch recreational skiers and analysed the level of overestimation. The authors found that male beginners and slightly advanced skiers who skied the least hours per day overestimate their abilities (10). To reduce the risk of accidents and fatalities it is important that skiers can assess their skiing skills. However, Sulheim et al. (11) showed that skiing ability self-assessment is difficult. The authors found in their study of 512 alpine skiers, Telemark skiers, snowboarders, and skiboarders, low to fair correlations between self-reported and expert skiing skills (11).

To capture human motion in-field and to give feedback to athletes or coaches smart equipment and sensor systems can be used. Inertial Measurement Units (IMUs) and other wearable sensor systems have already been applied in many areas for sports and performance analysis [see e.g., the review of Seçkin et al. (12)]. In alpine skiing, IMUs can be used to give skiers or coaches insights on kinematic parameters [e.g., (13)] and to support them during training sessions [e.g., (14)]. Feedback on skiing ability is not only interesting for professional athletes, but also for amateurs and recreational skiers to analyse e.g., the turning skills (15). To provide feedback on skiing performance various prototypes, such as the SmartSki of Kos and Umek (16) have already been developed for recreational and professional skiers that measures forces applied to and bending of the skis, as well as motion parameters, such as acceleration and angular speed.

To analyse skiing quality and give skiers metrics related to their skiing abilities the Connected Boot (Atomic, Altenmarkt, Austria) was developed. The Connected Boot is a sensor based skiing quality analysis tool, which is based on an IMU-sensor strap that is mounted on the skiing boot and which was developed in various research cycles (17–24). In addition, the Connected Boot was used to detect big air jumps and jumps during skiing (25). The Connected Boot assesses skiing quality and gives feedback on the skiing technique via skiing relevant parameters, such as the speed, the edge angle or the skiing quality with a score between one and 10, that assesses the carving skills. According to Jentschura and Fahrbach (26), carving is the term used to describe a turn in which only the edges of the ski and not the ski surface describe the skier’s trajectory. Furthermore, according to the authors, carving must not involve slipping or grinding.

The skiing quality score was developed and validated using data from experienced skiers in the field by Snyder et al. (24) and has not yet been tested in a larger study with differently characterised skiers and recreational skiers.

Therefore, the aim of this study was to analyse whether the skiing quality score delivers reasonable results for recreational skiers. We analysed whether self-assessed skiing ability corresponds to the objectively assessed skiing quality, determined by calculated metrics of the Connected Boot and investigated how the self-assessment changes before and after the study when the participants received the skiing relevant parameters via the Connected Boot app. For self-assessment the skiers received a questionnaire at the beginning, when they received the sensor system and one at the end, when they finished their last run. Data collection run from February to April 2023 in an own chosen skiing resort. Using a living lab approach, the testing was conducted in a real-life setting, where entire cities or areas (e.g., slopes) function as spaces for data collection or testing with real users (recreational skiers). The living lab concept emphasizes user-centered innovation and co-creation, which involves active user participation in the innovation and development process (27).

## 2 Materials and methods

Data for the analysis were collected with an IMU-based sensor system (the Connected Boot strap), as well as with questionnaires, that the participants had to fill out when they received and returned the sensor system.

### 2.1 Connected boot sensor system

**Figure 1:**
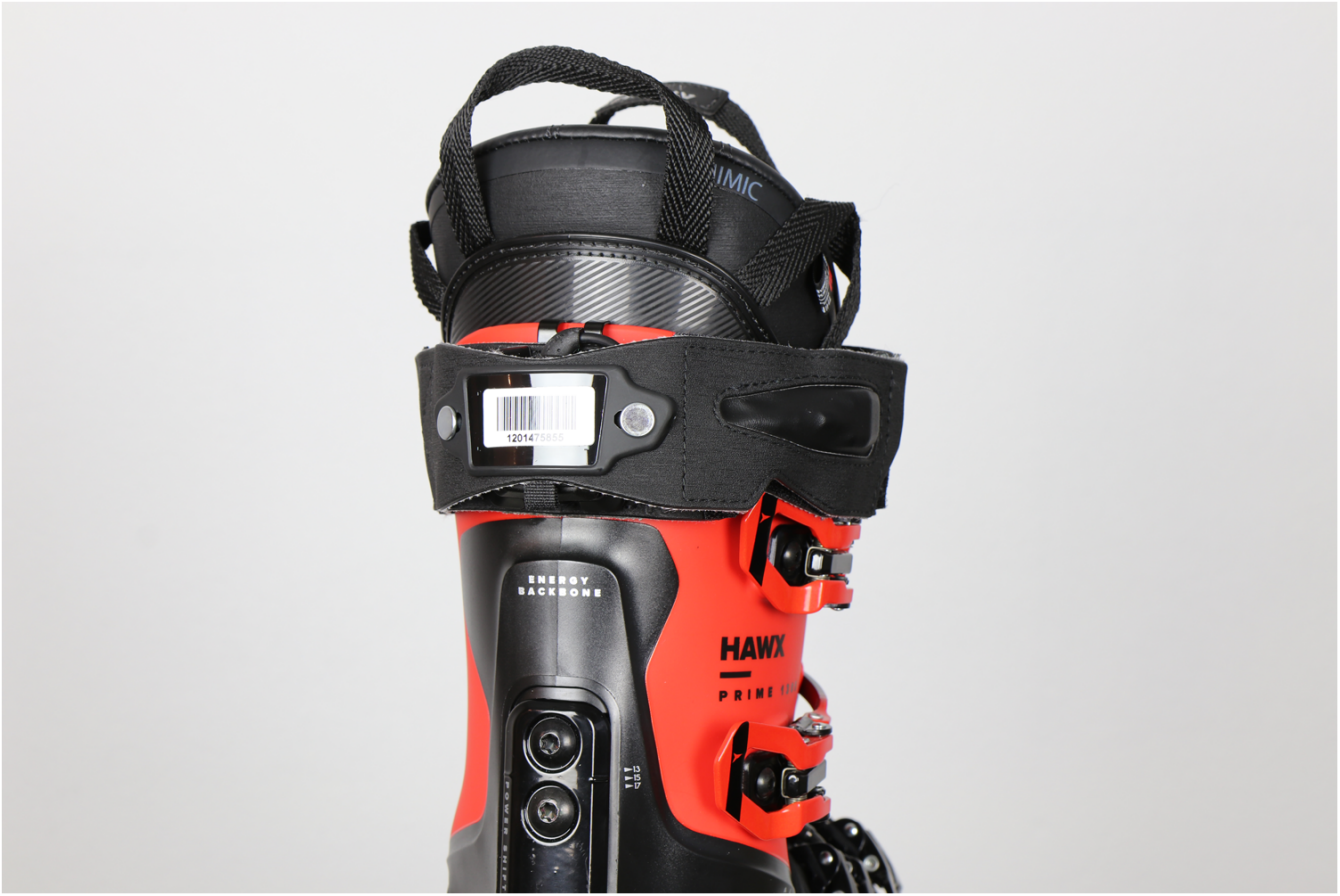
Connected Boot with strap.

The Conneted Boot is a sensor system that gives feedback on the skiing performance. It was developed within various research cycles and started with laboratory and infield measurements to identify the best sensor set-up and to develop first algorithms, such as a turn detection algorithm that was validated in the laboratory (19), as well as in-field (18). In addition to the recognition of ski turns, the system gives feedback on skiing specific parameters, such as on the general skiing style [snowplow, snowplow steering, carving or drifting, (20)] or on motion quality parameters, such as information on edge angle or radial forces (24). With regard to skiing quality analysis, the Connected Boot displays a motion quality score, developed by Snyder et al. (22) that is based on Principal Component Analysis. The Connected Boot consists of two IMUs (configuration: 2.5×3×0.83 mm ±16 g and ±1000 dps full-scale resolution, board by Movesense). The system is attached to the upper posterior cuff of the ski boot as visualized in Figure 1. The accelerometer and the gyroscope signals are sampled at 833 Hz and filtered with a low pass filter (cutoff: 416.5 Hz–accelerometer; 245 Hz–gyroscope). After filtering, data were then transmitted via Bluetooth to smartphones, further processed and the results displayed in an app (see Section 2.2). To make the app accessible to as many people as possible, there were two versions (an Android and an iOS version). Filtered data were transmitted at 54 Hz on iOS smartphones and 33 Hz on Android smartphones.

### 2.2 App

**Figure 2:**
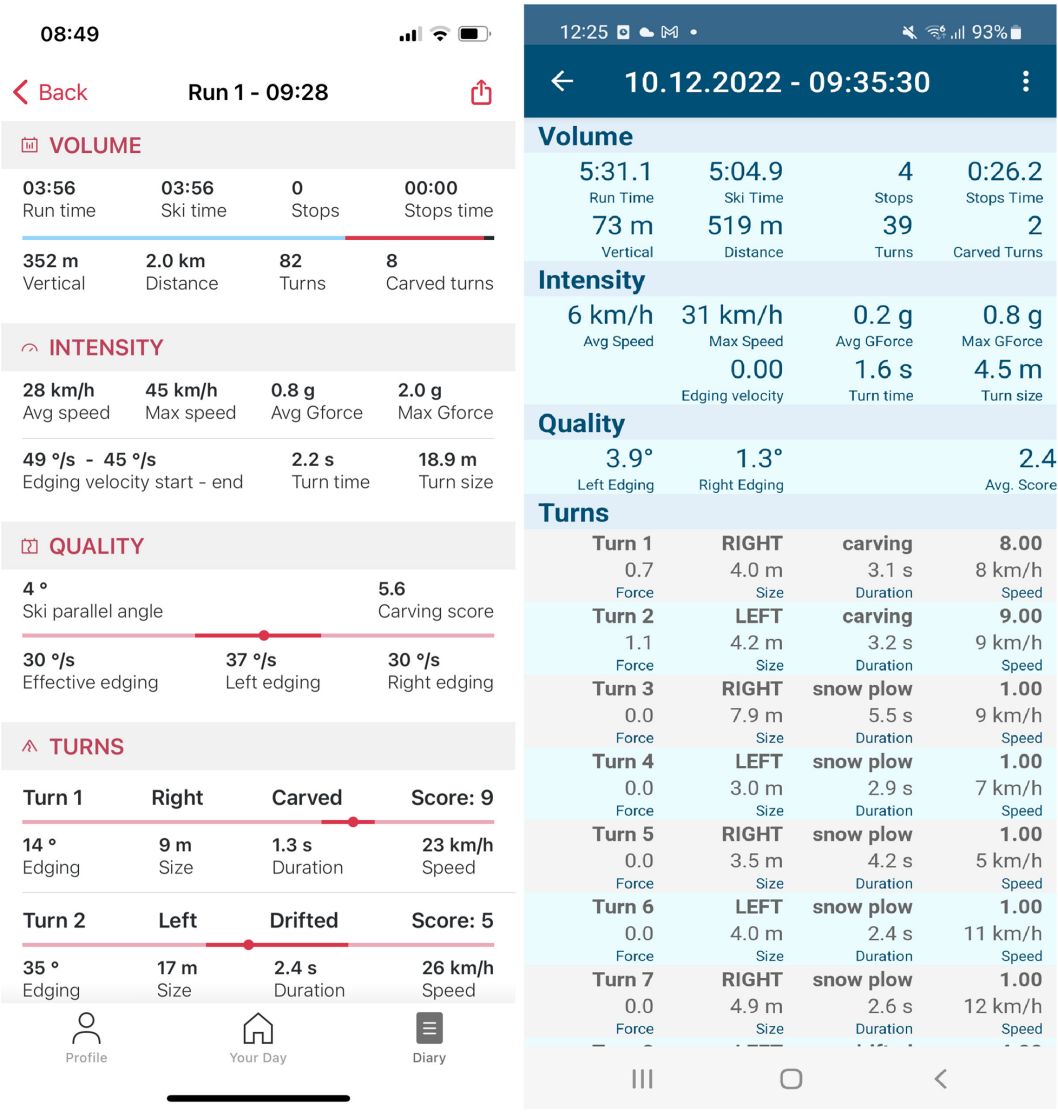
Screenshots of the iOS (left screenshot) and Android app (right screenshot).

**Figure 3:**
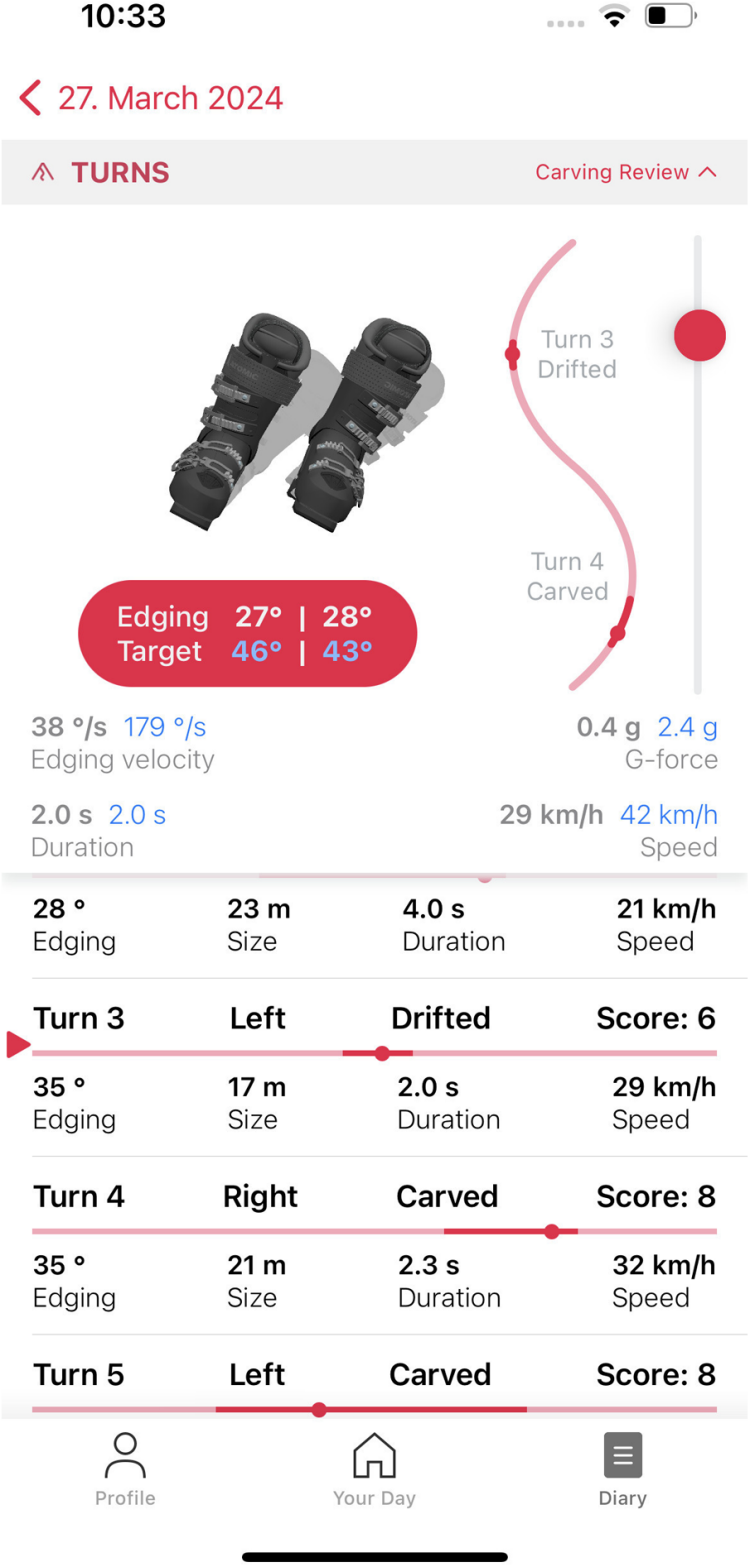
Exemplary user feedback for edging when skiing.

Both versions of the app display various parameters that were collected and calculated during a ski run. Parameters include turn size (radius) in meters, turn duration, speed (average and maximum), g-forces, as well as edge angle, and in the case of iOS, edge angle velocity (the speed at which the maximum angle was reached during a turn). In addition to this, the app shows a new metric, developed by Snyder et al. (22) that measures the skiing quality via a score, ranging from one to 10 from snow-plow to snow-plow-steering to drifting to carving. Figure 2 shows screenshots of the two versions of the app. They display aggregated details on the total run (e.g., average speed), as well as detailed information on each turn (e.g., turn style). In addition to the ski quality score, another useful function of the app that gives the skier direct feedback is the visual comparison of the ideal and actual edging for each individual ski turn. Figure 3 shows an example of this for a selected ski turn. The shadow shows the ideal edging when skiing, while the black ski boots show the actual edging during the turn.

### 2.3 Data collection

All participants signed the consent form approved by the ethics committee of the Paris Lodron University Salzburg (EK-GZ 2/2023) and provided written informed consent before their study participation.

The study was conducted from February to April 2023. Based on the living lab concept, participants were able to select a ski resort of their choice and decide how often they wanted to use the system. Before the test started the participants received the Connected Boot strap.

For this study, a user-centric living lab approach was chosen: It is characterised by the fact that research prototypes or novel sensor systems - such as the Connected Boot - are tested in a real-life setting without clear instructions for users (28). This approach helps to gain authentic user feedback, identify design pain points, and evaluate the system’s learnability to support the iterative improvement of the system (29). The lack of a one-size-fits-all approach reflects the inherent flexibility of the living lab approach, which is essential for fostering genuine co-creation and feedback and addressing the unique requirements of each project. This adaptability, while potentially posing challenges in terms of consistency, ultimately empowers users to play a central role in developing innovative solutions that meet their needs and preferences (30). This in turn provides researchers and developers with important insights for product development, which positively impacts market entry (31).

For data collection the participants had to pair the sensors with their smartphones via Bluetooth (either Android or iOS) and to mount it centrally on the back of the ski boot (see Figure 1). Further, the participants were instructed to install either the Android or the iOS version of the Connected Boot app depending on their smartphones. At the beginning of the first run the participants started the app, made sure that GPS and Bluetooth were activated and that the sensors were paired with the smartphone. If there were several devices nearby, the participants were asked to scan the QR code on the back of the module. To start recording the run, participants should click on “start run”. Depending on the duration of the participants’ ski trip, they could let the app run throughout the whole trip or pause the recording (e.g., during cable car rides or breaks). The data collected on the device was periodically synchronized using a secure REST connection with an application server to (1) allow the app to recover data in the case of local data loss or re-install, and (2) to allow further data analysis and improvement of the algorithms and the app.

In total, 130 participants were enrolled in the study and received the sensor system. Of these 130 participants 51 were excluded because they either did not fill out the informed consent or (parts) of the survey. In the end, 62 of the remaining 79 could be included in the study, as data recordings were available with the strap and sensor data could be merged with the questionnaires. Table 1 shows an overview of the 62 participants, including the sample size and mean age by gender. The sample consisted of heterogeneous skiers in terms of age and self-assessed skiing skill level.

**Table 1:** Sample size and mean age of participants by gender.

Gender	n	Mean age
Female	16	42.4 (sd: 16.5)
Male	46	48.1 (sd: 13.6)
Diverse	0	–

In addition to the Connected Boot sensor system, the participants received a link to a questionnaire at the beginning of the study, which, in addition to demographic questions, included questions on general technology acceptance and the use of fitness trackers as well as their own assessment of their skiing skills (beginner, intermediate, advanced and expert), carving ability (score from one to 10) and skiing frequency. After the last run of their individual data collection the participants had to fill out a second questionnaire that contained questions about the user experience of the Connected Boot and again a question on their carving ability as a score between one to 10.

The questionnaire was designed to investigate the user acceptance of the Connected Boot system, using the Technology Acceptance Model (TAM) as the scientific framework (32, 33). It covers several key aspects to assess the technology acceptance, such as perceived usefulness of the Connected Boot in tracking their skiing skills and improving their performance. It also examines the perceived ease of use, evaluating how easy it is for users to learn and become skilled at using the system. Additionally, the questionnaire measures the users’ behavioural intention to use the Connected Boot in the future, which is a crucial indicator of technology acceptance (33). The questionnaire also explored other relevant factors, such as self-efficacy, self-monitoring, and perceived enjoyment (34).

The study ended for each participant by returning the equipment.

### 2.4 Data analysis

In a preprocessing step, skiing quality data, collected via the Connected Boot, was merged to the questionnaire data. Further, data of each run were aggregated per participant and filtered. Skiing quality scores of zero, which can be interpreted as unrecognized turns due to going straight or problems with the sensor connection or turn detection, were filtered out. All runs that were shorter than 100 meters and 10 s were also not considered. To analyse the data exploratory data analysis, statistical hypothesis tests and correlation analyses (Wilcoxon signed-rank test, Wilcoxon rank sum test, Kruskal–Wallis test [incl. post-hoc analysis with p-value adjustment (35)] and Spearman correlations) were used. For all tests the level of significance was set to alpha = 0.05. For interpretation, we follow the suggestion of Cohen (36) and interpret a correlation between 0.1 and 0.3 as weak, a correlation between 0.3 and 0.5 as moderate and larger than 0.5 as strong correlation. Data preprocessing, data analysis, visualizations and statistical tests were carried out with R (37).

## 3 Results

### 3.1 Overview and descriptive analysis

Merging all the Connected Boot data with the questionnaires was possible for in total 62 skiers, 15 were Android and 47 iOS users. Due to the different versions and brands of Android smartphones, there were more technical problems with the Android app than with the iOS app. Of these 62 participants 16 (25.81%) were female and 46 (74.19%) male. The age ranged from 16 to 72 years, with a mean of 46.6 (sd = 14.5). Two (3.23%) of them rated themselves as beginner, two (3.23%) as intermediate, 31 (50.00%) as advanced and 27 (43.55%) as expert skiers.

Six (9.68%) of the participants stated to ski up to seven days per season, 18 (29.03%) to ski between eight and 15 days and 38 (61.29%) to ski more than 15 days. In terms of gender, all male skiers classified themselves as advanced or expert skiers, while a quarter of female skiers classified themselves as beginners or intermediate skiers (see Table 2).

**Table 2:** Level of skiing and number of general skiing days (outside the study), grouped by gender.

	Female	Male
Skill level		
Beginner	2 (12.50%)	0 (0.00%)
Intermediate	2 (12.50%)	0 (0.00%)
Advanced	8 (50.00%)	23 (50.00%)
Expert	4 (25.00%)	23 (50.00%)
General skiing frequency		
Up to 7 days	3 (18.75%)	3 (6.52%)
8 to 15 days	6 (37.50%)	12 (26.09%)
More than 15 days	7 (43.75%)	31 (67.39%)

During the study 35 (56.45%) of the participants used the Connected Boot on one day and 27 (43.55%) on more than one day. From these 27 who used it more than once 14 (22.58%) participants skied on two, seven on three days (11.29%), three (4.84%) on four days. In addition, one person (1.61%) used the system on six, 10 and 17 days, respectively (mean = 2.08 days, sd = 2.42). The number of runs per person varied from one to 506.

When the participants returned the system, 50 (80.64%) completely or mostly agreed, six (9.68%) completely or mostly disagreed and six (9.68%) were indifferent that they received useful information from the Connected Boot sensor system. A majority of participants, 42 individuals (67.77%), completely or mostly agreed that they tried to improve their skiing style while using the Connected Boot. In contrast, 12 participants (19.35%) completely or mostly disagreed, and eight (12.90%) expressed indifference. In addition, 19 (30.65%) participants completely or mostly agreed that they were able to improve their skiing style by using the Connected Boot, 18 (29.03%) were indifferent and 25 (40.32%) completely or mostly disagreed. Furthermore, 44 (70.97%) completely or mostly agreed to use the system in future, 11 (17.74%) were indifferent and seven (11.29%) completely or mostly disagreed to use it in future (see Table 3).

**Table 3:** Acceptance of the Connected Boot sensor system.

	Disagree	Mostly disagree	Indifferent	Mostly agree	Agree
I have gained useful information about my skiing style	1 (1.61%)	5 (8.06%)	6 (9.68%)	25 (40.32%)	25 (40.32%)
I tried to improve my skiing style while testing the Connected Boot	5 (8.06%)	7 (11.29%)	8 (12.90%)	19 (30.65%)	23 (37.10%)
By using the Connected Boot, I was able to improve my skiing style	13 (20.97%)	12 (19.35%)	18 (29.03%)	16 (25.81%)	3 (4.84%)
I would use the Connected Boot in the future	2 (3.23%)	5 (8.06%)	11 (17.74%)	25 (40.32%)	19 (30.65%)

### 3.2 Skiing quality assessment

To assess whether the skiing quality score delivers reasonable results for recreational skiers we analysed the correlation between the self-reported skiing quality (via questionnaires) and the objective measured skiing quality score.

#### 3.2.1 Objective skiing quality score of the Connected Boot

**Figure 4:**
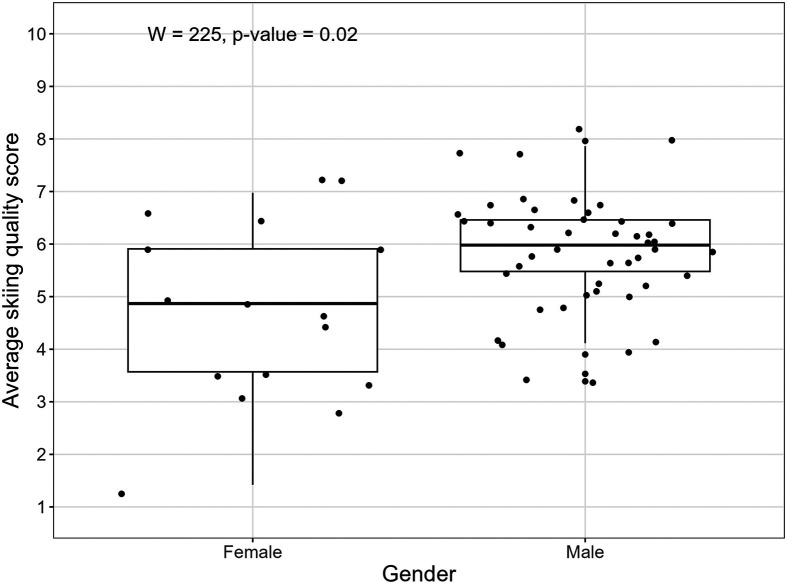
Mean skiing quality score per participant, grouped by gender.

The average objective skiing quality score collected with the Connected Boot of all runs and participants was 5.55 (sd = 1.32). The mean score over all runs per participant ranged from 1.42 to 7.96. As also visualized in Figure 4 the mean skiing quality score of female participants was with 4.72 (sd = 1.65) statistically significant (Wilcoxon rank sum test p=0.02) lower than the skiing quality score of male participants (5.84, sd = 1.06). When analysing the skiing quality score in relation to the participants’ age no significant difference could be found (Kruskal–Wallis rank sum test p=0.72). Skiers who stated to ski more than 15 days achieved significantly higher (Kruskal–Wallis rank sum test p<0.001) mean skiing quality (mean = 5.93; sd = 1.14) scores than skiers who skied up to seven days (mean = 4.10, sd = 0.97) or eight to 15 days (mean = 5.23; sd = 1.41).

Between the mean skiing quality score and the self-reported skiing skill level (beginner, intermediate and expert) a tendency towards a significant difference was found (Kruskal–Wallis rank sum test p=0.06). The average skiing quality score of beginner skiers was 3.32 (sd=0.49), of intermediate skiers 4.10 (sd=0.60), of advanced skiers 5.76 (sd=0.87) and of expert skiers 5.59 (sd=1.61).

#### 3.2.2 Self-assessed skiing quality score

**Figure 5:**
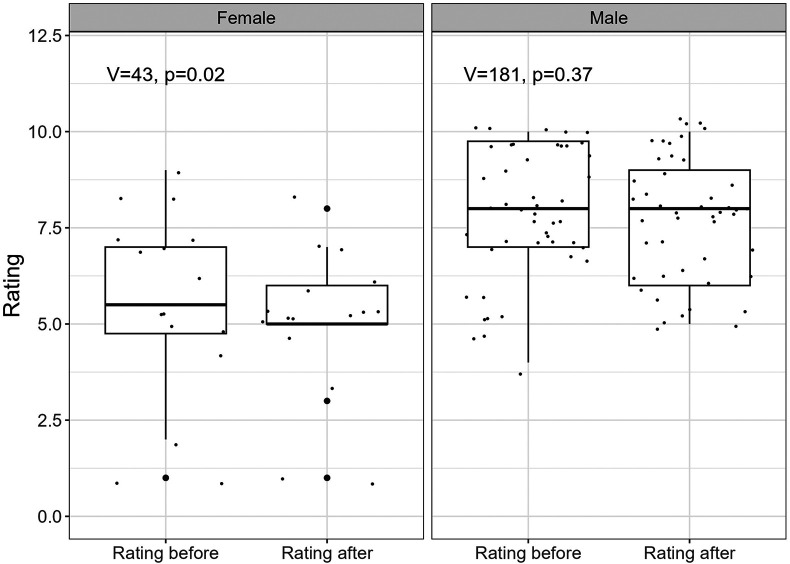
Subjective rating before and after the study, grouped by gender.

The mean self-reported carving ability score was 7.26 (sd=2.19) before the study and 6.98 (sd=2.08) after the study, when returning the sensor system. Females reported a mean carving ability score of 5.44 (sd=2.45) before and 4.94 (sd=1.91) after the study. Male skiers reported a mean score of 7.89 (sd=1.72) before and 7.70 (sd=1.63) after the study. The mean difference between the beginning of the study and the end was 0.27 (sd=1.38). 28 (45.16%) participants did report the same carving ability score, 15 (24.19%) a smaller score of that at at the beginning and 19 (30.65%) a larger score. Figure 5 plots the stated carving skill score before and after the study, grouped by gender.

#### 3.2.3 Correlation between the objective skiing quality score and the self-assessed carving ability

**Figure 6:**
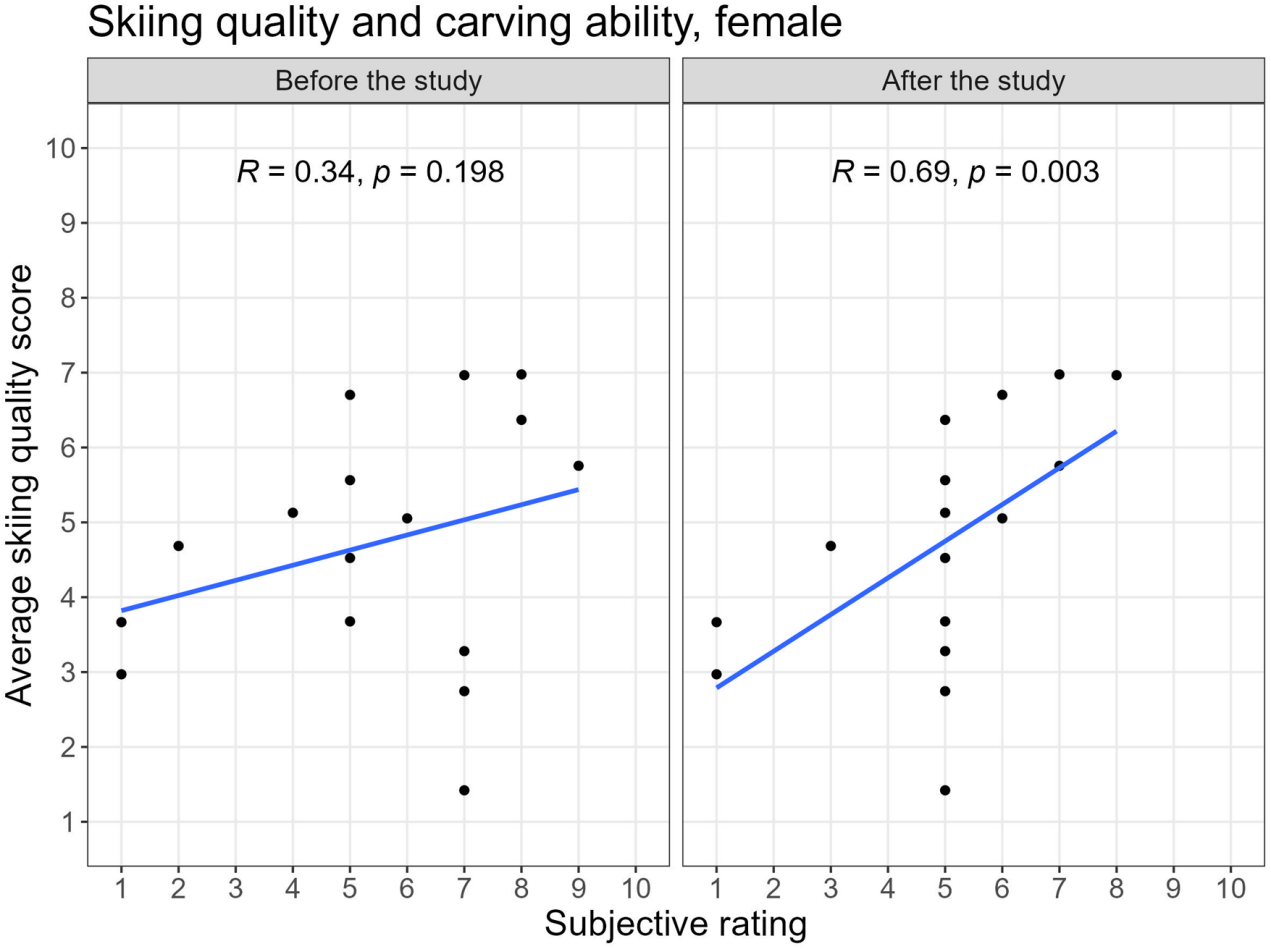
Correlation between subjective stated carving ability and objective skiing quality score before and after the study, female.

**Figure 7:**
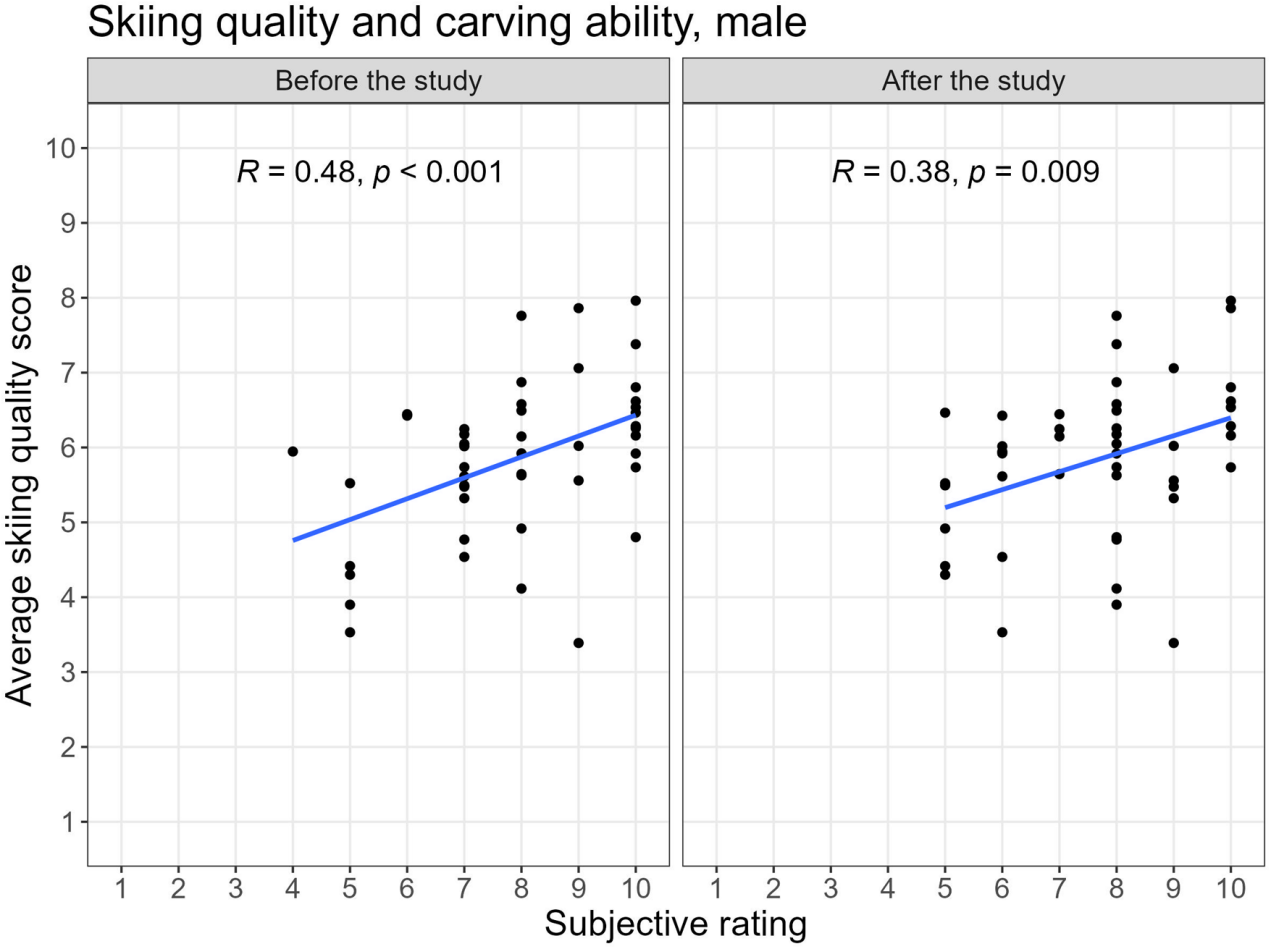
Correlation between subjective stated carving ability and objective skiing quality score before and after the study, male.

Overall, the Spearman correlation between the self-reported carving ability and the skiing quality score of the Connected Boot was of 0.52 (p<0.001) both at the beginning and at the end of the study for all participants together. However, there were difference on a gender-level: while the correlation between the subjective carving ability and the skiing quality score of the Connected Boot was not significant (0.34, p=0.20) before the study, it increased to 0.69 (p<0.001) after the study for female skiers. For male skiers the correlation between the self-reported carving ability and the skiing quality score of the Connected Boot decreased from 0.48 (p<0.001) before to 0.38 (p=0.01) after the study. Figures 6 and 7 show the correlation between the subjectively reported carving ability and the ski quality rating of the Connected Boot before and after the study for each gender.

## 4 Discussion

### 4.1 Main results

This study analysed the skiing quality of recreational skiers, calculated with the Connected Boot sensor system, in a living lab setting. Participants skied without any instructions at various self-selected skiing resorts. While the majority of the participants agreed that the Connected Boot sensor system provided useful information about skiing, many were indifferent or disagreed that they could improve their skiing style during the study. One reason for this may be the varying and sometimes very short duration with a mean of 2.08, a minimum of one to a maximum 17 days for which the system was used.

The self-reported skiing quality before the study with an average score of 7.26 (sd = 2.91) was 1.71 points higher than the algorithm-based skiing quality score with an average score of 5.55 (sd = 1.32). However, as the calculated skiing quality score takes into account all runs over the day, which also includes runs on full pistes or pistes with bad snow conditions where it is difficult to carve unlimited with high speed, this difference between the two values seems to be plausible.

According to Cohen (36), the strong correlation of 0.52 between the self-reported carving ability before the study and the skiing quality score from the Connected Boot indicates that the skiing quality algorithm provides reliable results, even for recreational skiers.

However, differences in the skiing quality score were found between male and female skiers, where male skiers achieved on average higher scores than female skiers. A differentiated analysis by gender shows genders-specific differences in the capacity to assess skiing abilities. The analysis of the pre and post study self-assessed carving ability showed that the correlation coefficient between the self-assessment and the skiing quality score was not significant before the study and increased to 0.69 for female skiers after the study, whereas it decreased for male skiers from 0.48 to 0.38. For male skiers the correlation between the self-assessment and the skiing quality score can be interpreted as a moderate correlation before and after the study. The correlation for female skiers increased from not significant before to a strong correlation after the study. Therefore, it seems that the female skiers reacted to the feedback from the Connected Boot app and adjusted their subjective assessment of their carving ability accordingly, whereas the male skiers did not adapt. Possible explanations for the low correlation coefficient of men could be different learning curves or adaptation speeds in relation to the feedback from the Connected Boot due to differences in the motivation, attitude or experience of the participants. Although these results have to been interpreted when taking into account the small number of female participants, these findings go in line with the results of Roberts and Nolen-Hoeksema (38) who found that feedback of others influence men and women self-assessment of abilities in performance situations in different ways. Especially females find the assessment of others informative and are influenced by positive, as well as negative feedback (38). Also the meta-analysis of Kim and Cruz (39) showed that there are gender differences in the reaction of coach feedback, as female players exhibited greater improvements in satisfaction and commitment in response to transformational leadership compared to male players.

### 4.2 Limitations and outlook

The aim of the study was to analyse the objective and subjective skiing quality score of recreational skiers, who went skiing independently in their own chosen skiing resorts, based on a living lab concept. Apart from the instructions for installing and using the Connected Boot, the participants received no further information and were unobserved. Therefore, some skiers only used the system for one day, which is a limiting factor in the analysis of the change of self-assessment. In order to test whether the sensor system could be made to work by the recreational skiers themselves, we decided not to provide them with any experts and plan the study in form of a living lab concept. The study followed a not-standardized methodology, using a user-centric and open living lab approach (different slopes, different time of usage). Although the living lab approach still receives little attention in the development of novel sport technologies and research on it is still limited, many insights for future living lab studies have been gained. It is a promising method for involving end users in the feedback loops as early as the research and development process and shortening the research-to-market time. The focus on users’ technology acceptance in the early development stage can prevent wrong decisions and developments and lead to widely applied products. However, this approach led to some people reporting technical difficulties and the resulting lack of data recordings and a reduced number of participants who could be included in the data analysis.

For the analyses the mean skiing quality score calculated across all runs and turns was used for each participant, as these values are also shown in the Connected Boot app. However, it should be noted that all ski turns longer or equal than 10 s and 100 meters, including those directly after or before the lift runs, were taken into account. As the calculation of the skiing quality score is based on various parameters, such as skiing speed or edge-angle, the turns directly after lift runs will have smaller skiing quality score values due to the e.g., slow speed. Therefore, the mean skiing quality score is around eight also for the best skiers of the study. In general, skiing quality is influenced by many factors. Participants especially reported weather and slope conditions, as well as other skiers on the skiing piste as crucial factors to be able to carve freely. To achieve a higher mean skiing quality score, a different setting, such as a closed-off piste, would therefore be necessary. On the other hand this also weakens the external validity in the ecological environment of Alpine skiing. As we analysed in this pilot study the self-assessment of the skiing skills and compared these values with the skiing quality score of Snyder et al. (22) future research could also include and analyse expert assessment of the skiing skills via video labeling, apart from the subjective stated assessment. Initially, we also planned to compare the results of the skiing quality score with the assessment by ski instructors. A ski school and a group of beginner skiers were selected, but due to the amount of time required for the visual assessment, the ski instructors had to cancel the assessment as normal ski school operations could not be maintained. Future studies should therefore take into account the high amount of time required for a visual and objective assessment by experts and endeavour to obtain a strong commitment from the participants. Futhermore, future studies should also systematically investigate the continuous use of the sensor system. In order to explain the different correlation coefficients between the subjectively reported carving quality and the objective ski quality between female and male participants, future studies should involve larger sample sizes, more details on participants’ background information, and qualitative interviews to capture motivational and attitude-related factors. For future app development, the differences in the interpretation of the app feedback by different user types could be taken into account. For example, specific cluster groups could be identified first, allowing feedback to be tailored to each respective type.

## 5 Conclusion

The aim of the pilot study was to investigate the objectively assessed skiing quality and the self-assessment of skiing skills of recreational skiers who went skiing independently in form of a living lab concept. The goal was to assess whether the self-assessed skiing quality score corresponded to the objectively assessed score and whether the correlation changed from the start of the study, when the participants received the sensor systems to the end, when they returned it. Future studies should further analyse a longer and continuously use of the Connected Boot sensor system and analyse the development of the skiing skills within these long-term study. The results showed that male skiers achieved a higher mean skiing quality score than female skiers and also skiers who stated to ski more frequently achieved higher scores than skiers with less skiing days. Further, while the correlation between the quality score of the Connected Boot and the self-assessed carving ability increased from the beginning compared to the end of the study for female skiers, it decreased for male skiers. This suggests that the female skiers were more likely to accept the feedback from the app and adapt the assessment of their ability.

## Data Availability

The raw data supporting the conclusions of this article will be made available by the authors, without undue reservation.
